# Vitamin D Fortification of Milk Would Increase Vitamin D Intakes in the Australian Population, but a More Comprehensive Strategy Is Required

**DOI:** 10.3390/foods11091369

**Published:** 2022-05-09

**Authors:** Eleanor Dunlop, Anthony P. James, Judy Cunningham, Anna Rangan, Alison Daly, Mairead Kiely, Caryl A. Nowson, Paul Adorno, Paul Atyeo, Lucinda J. Black

**Affiliations:** 1 Curtin School of Population Health, Curtin University, Kent Street, Bentley, WA 6102, Australia; eleanor.dunlop@curtin.edu.au (E.D.); t.p.james@curtin.edu.au (A.P.J.); judyc121@gmail.com (J.C.); alison.daly@curtin.edu.au (A.D.); 2 School of Life and Environmental Sciences, The University of Sydney, Camperdown, NSW 2006, Australia; anna.rangan@sydney.edu.au; 3 Cork Centre for Vitamin D and Nutrition Research, School of Food and Nutritional Sciences, University College Cork, T12 K8AF Cork, Ireland; m.kiely@ucc.ie; 4 Institute for Physical Activity and Nutrition Research, Deakin University, 221 Burwood Highway, Burwood, VIC 3125, Australia; caryl.nowson@deakin.edu.au; 5 National Measurement Institute, 1/153 Bertie Street, Port Melbourne, VIC 3207, Australia; paul.adorno@measurement.gov.au; 6 Australian Bureau of Statistics, 45 Benjamin Way, Belconnen, ACT 2617, Australia; paul.atyeo@abs.gov.au; 7 Curtin Health Innovation Research Institute (CHIRI), Curtin University, Bentley, WA 6102, Australia

**Keywords:** Australia, food, fortification, milk, vitamin D

## Abstract

Low vitamin D status (serum 25-hydroxyvitamin D (25(OH)D) concentration < 50 nmol/L) is prevalent in Australia, ranging between 15% and 32% in the adolescent and adult populations. Vitamin D intakes are also low across the population and were recently estimated at 1.8–3.2 µg/day on average, assuming equal bioactivity of the D vitamers. In combination, these findings strongly suggest that data-driven nutrition policy is needed to increase vitamin D intake and improve status in the Australian population. Food fortification is a potential strategy. We used up-to-date vitamin D food composition data for vitamin D_3_, 25(OH)D_3_, vitamin D_2_, and 25(OH)D_2_, and nationally representative food and supplement consumption data from the 2011–2013 Australian Health Survey, to model a fortification scenario of 0.8 µg/100 mL vitamin D for fluid dairy milks and alternatives. Under the modelled fortification scenario, the mean vitamin D intake increased by ~2 µg/day from baseline to 4.9 µg/day from food only (7.2 µg/day including supplements). Almost all individual intakes remained substantially below 10 µg/day, which is the Estimated Average Requirement in North America. In conclusion, this modelling showed that fortification of fluid milks/alternatives with vitamin D at the current permitted level would produce a meaningful increase in vitamin D intake, which could be of potential benefit to those with a low vitamin D status. However, this initial step would be insufficient to ensure that most of the population achieves the North American EAR for vitamin D intake. This approach could be included as an effective component of a more comprehensive strategy that includes vitamin D fortification of a range of foods.

## 1. Introduction

Mean usual intakes of vitamin D from food in the Australian population were estimated to range from 1.8 to 3.2 µg/day, assuming equal bioactivity for vitamin D and 25-hydroxyvitamin D (25(OH)D) [1]. This is considerably lower than the Institute of Medicine’s (IOM) Estimated Average Requirement (EAR) of 10 µg/day [2], which is used here in the absence of an Australia-specific EAR [3]. While the IOM’s EAR assumes minimal sun exposure, there is a relatively good opportunity for sun exposure in Australia. However, the year-round prevalence of low vitamin D status (serum 25-hydroxyvitamin D (25(OH)D) concentration < 50 nmol/L [4]), which is associated with unfavourable skeletal outcomes, including bone fracture and bone loss, ranges between 15% and 32% in the Australian adolescent and adult populations [5,6]. This prevalence is higher still (~40%) for remote-dwelling Aboriginal and Torres Strait Islander adults [7]. In winter, the prevalence of low vitamin D status increases to 40 and 50% in states at higher latitudes (Victoria, South Australia, New South Wales, Australia Capital Territory, and Tasmania) [8]. Paired with low intakes of vitamin D, these data suggest that data-driven nutrition policy is needed to improve the population’s vitamin D status. 

In other countries, the dietary supply of vitamin D has been increased through (largely voluntary) fortification of popular foods, such as milk, orange juice, bread, breakfast cereals, rice, pasta, and noodles [9,10,11,12,13,14,15,16]. In Finland, the systematic fortification of fluid milk products and edible oil spreads in 2003, alongside voluntary fortification of other foods, such as bread, juice, and mineral water, reduced the prevalence of 25(OH)D below 50 nmol/L from 56 to 9% by 2011 in non-consumers of vitamin D-containing supplements [13]. In Australia, edible oil spreads must be fortified with a minimum of 5.5 µg/100 g vitamin D [17], and few other foods are voluntarily fortified with vitamin D. These differences in vitamin D fortification practices may explain the lower usual intakes of vitamin D reported for the Australian population [1] compared with those reported for the US, Canada, and some European countries [14,18,19,20,21]. Furthermore, the difference between usual intakes of vitamin D in Australia and other countries may be greater still since four D vitamers were accounted for across all food types when estimating usual vitamin D intakes in Australia, which has not been the case for estimates made for Canada and the US [22,23,24].

Fluid milk products and their alternatives (e.g., soy milk and other plant-based alternatives) are among the few foods that are permitted for vitamin D fortification in Australia. As an initial step to provide data to address this problem, we modelled the effect of vitamin D fortification of all fluid milks/alternatives at the maximum permitted level on vitamin D intake.

## 2. Materials and Methods

### 2.1. Study Population and Food Consumption Data

The 2011–2013 National Nutrition and Physical Activity Survey (NNPAS) was an arm of the 2011–2013 Australian Health Survey (AHS), which is the most recent nationally representative health survey conducted in Australia. Trained interviewers from the Australian Bureau of Statistics collected food and dietary supplement consumption data for participants of the NNPAS (*n* = 12,153, aged ≥ 2 years). Detailed descriptions of the methods used are available elsewhere [1,25]. Briefly, the Automated Multiple-Pass Method [26], modified to represent foods available in Australia, was used to collect food consumption data through two 24 h dietary recalls. The Day 1 dietary recall was conducted in person and was completed by 12,153 NNPAS participants; the Day 2 dietary recall was conducted by telephone call (scheduled ≥8 days after Day 1 and on a different day of the week) and was completed by 64% (*n* = 7735) of participants. The AHS was conducted in accordance with the Declaration of Helsinki. NNPAS interviews were conducted under the Census and Statistics Act 1905.

### 2.2. Vitamin D Content of Foods for the Baseline Model

We used up-to-date comprehensive analytical vitamin D food composition data [27] to map vitamin D concentrations in 5740 foods in AUSNUT, Australia’s survey food composition database [28]. The methods are described in greater detail elsewhere [1]. Briefly, 98 different food products (149 analytical samples from 896 primary samples) were purchased in Sydney, Melbourne, and Perth in 2018 and 2019. Samples were prepared as they would be consumed [27]. Four D vitamers (vitamin D_3_, 25(OH)D_3_, vitamin D_2_, and 25(OH)D_2_) were measured using a validated liquid chromatography with triple quadrupole mass spectrometry method (ISO17025:2017) at the National Measurement Institute of Australia. Vitamin D equivalents (VDE) were calculated for each food product as the sum of the analytical concentrations of the four D vitamers, assuming equal bioactivity [29]. VDE concentrations were mapped to AUSNUT food entries according to methods used previously for Australian total diet studies [30,31]. AUSNUT foods (*n* = 5740) can be classified as non-recipe foods (consisting of a single ingredient) or recipe foods (consisting of multiple ingredients). We manually assigned VDE concentrations to non-recipe foods. Food Standards Australia New Zealand’s custom dietary modelling software, Harvest [32], was used to assign vitamin D concentrations across multiple levels of recipes for recipe foods. Where necessary, conversion factors were used to adjust concentrations for foods reconstituted from a dehydrated form (e.g., infant formula) [30]. Similarly, a conversion factor was used to derive concentrations from a related food (e.g., estimating a concentration for orange juice from the VDE value for oranges).

### 2.3. Adjusting the Vitamin D Content of Foods for the Fortification Model

Baseline vitamin D concentrations of AUSNUT entries for fluid milks/alternatives were adjusted to reflect a fortification concentration of 0.8 µg/100 mL, which is the maximum permitted in Australia [17]. To capture relevant non-recipe foods, food names in the AUSNUT Nutrient Database [28] were searched for ‘milk’, which also captured plant-based milk alternatives. Flavoured milks/alternatives were assumed to contain 95% fluid milk, allowing for added sugar and flavourings. To capture relevant recipe foods, the AUSNUT Food Recipe File [28] was searched for ‘milk’. Vitamin D concentrations were assigned to recipe foods on the basis of the proportion of the fluid milk/alternative ingredient(s). As recipe foods may contain other recipe food items nested within them (e.g., the milk-based sauce recipe within a lasagne recipe), concentrations were assigned to capture this on the basis of the proportion of milk in the nested recipe food items. Milk used for other dairy production (e.g., cheese and yoghurt) was assumed to be diverted prior to fortification; hence, dairy products other than fluid milk/alternatives remained unfortified under this scenario. 

### 2.4. Vitamin D Content of Supplements

Vitamin D-containing supplements reported as consumed by participants of the NNPAS [25] included single vitamin D and multi-nutrient preparations, fish liver oils, and fish oils [33]. As described elsewhere [33], the Australian Register of Therapeutic Goods (ARTG) [34] was used to determine the vitamin D content of supplements. Where supplement composition data were not available from the ARTG, they were obtained directly from manufacturers.

### 2.5. Estimating Absolute Vitamin D Intakes for Baseline and Fortification Models

For each model, vitamin D concentrations for all AUSNUT foods were merged with Day 1 food and vitamin D-containing supplement consumption data for participants (*n* = 12,153) of the NNPAS [25] in Stata Statistical Software version 15 (StataCorp LLC, College Station, TX, USA). As the methods most suitable for estimation of usual intakes cannot process the multi-modal distributions of food and supplements [25], only Day 1 consumption data were used to estimate absolute intakes in order to capture the contribution of supplements. The mean, standard deviation, and range of estimated vitamin D intakes from food only and with supplements were reported for all participants and by the sex and age groups used in the Nutrient Reference Values for Australia and New Zealand [3]. 

## 3. Results

On Day 1 of the survey, 87% of participants consumed at least one fluid milk/alternative. Of 12,153 survey participants, 17% (*n* = 2039) reported taking a vitamin D-containing dietary supplement. With fortification of fluid milks/alternatives, mean vitamin D intake increased by ~2 µg/day from baseline to 4.9 µg/day from food only and to 7.2 µg/day with the inclusion of supplements (Table 1). In children aged 2–3 years, who had the lowest baseline intakes, mean vitamin D intake approximately doubled under the fortification model. The smallest increase occurred in females aged 51–70 years; however, as the mean baseline intake was comparatively high in this population group, this small increase resulted in a mean intake under the fortification model closer to the EAR of 10 µg/day with intakes from supplements included. Mean intake remained considerably lower than 10 µg/day for the majority of Australians, even with intakes from supplements included.

## 4. Discussion

Modelling the fortification of fluid milks/alternatives with 0.8 µg/100 mL vitamin D showed that vitamin D intakes may safely increase under this fortification strategy by ~2 µg/day across the population. However, under this fortification model, the mean intake of vitamin D remained below the North American EAR of 10 µg/day [2], even with intakes from dietary supplements included. Furthermore, it should be noted that intake of vitamin D at the EAR may address the dietary needs of only 50% of the population [2]. The highest increase was seen in young children, who also had the lowest baseline intakes; however, there are no nationally representative data on vitamin D status for Australian children aged <12 years to determine whether their lower vitamin D intakes correlate with poorer vitamin D status. The smallest increase was seen in females aged 51–70 years. This population group also had the second-highest prevalence (22%) of vitamin D-containing dietary supplement use [33]. The majority (16%) of vitamin D-containing dietary supplement users in this group reported taking a multi-nutrient preparation (daily dose median = 5 µg, range = 0.1–25 µg), while 4 and 3% took a single vitamin D (daily dose = 25 µg) or calcium/vitamin D supplement (daily dose median = 5 µg, range = 1.3–25 µg), respectively [33]. 

Our previous dose–response analyses [35] suggest that an additional 2 µg/day of vitamin D from fortified foods may provide a mean (95% confidence interval) increase in serum 25(OH)D concentrations of 4.5 (3.5, 5.6) nmol/L in adults and 6.9 (2.8, 11.0) nmol/L in children. Therefore, this is a meaningful increase in vitamin D intake that may particularly increase 25(OH)D among individuals with low baseline status, thereby improving the overall distribution in the population. 

These findings suggest that a wider range of foods would require fortification with vitamin D to accommodate dietary diversity, and/or the maximum permitted amounts for vitamin D in fortified foods would need to be increased to substantially increase vitamin D intake across the population. Similar findings have arisen from other modelling studies that have shown that the addition of vitamin D to a single or limited range of foods can result in limited reach across the population [14,15,36]. More diverse fortification strategies have been deemed necessary in other populations, e.g., in Canada, where it has been proposed that the mandatory level of vitamin D in milk and margarines should be increased (to 2 µg/100 mL and 26 µg/100 g, respectively) and the reach of vitamin D fortification extended via other food vehicles [37]. This approach was successful in Finland, where, in addition to the routine fortification of fluid milks and edible oil spreads with 1.0 and 20 µg/100 g vitamin D, respectively, certain other foods may be voluntarily fortified with vitamin D. A number of studies have demonstrated that carefully considered fortification strategies that include a range of fortification vehicles may safely increase the intake of vitamin D at the population level [38].

Currently, the maximum permitted amounts that may be added to fluid milks/alternatives and edible oil spreads in Australia are 0.8 µg/100 mL and 16 µg/100 g, respectively [17]. Hence, changes to the current regulations of the Australia New Zealand Food Standards Code would be required to support a strategy such as those used in Finland and proposed in Canada. In this study, we aimed to model a fortification scenario that would fit within existing regulations. Other widely consumed foods, such as bread, may be suitable for vitamin D fortification in Australia. Bread is already routinely fortified through the mandated use of iodised salt and bread flour fortified with folic acid and thiamin [17], but it is not yet permitted for vitamin D fortification. Future studies could simulate expanded fortification models to determine whether updates to the current regulations for vitamin D food fortification would be safe and appropriate for the Australian population.

A strength of our study was the use of nationally representative food consumption data and comprehensive food composition data for four D vitamers. However, since we used food consumption data from a single day, we were not able to assess the adequacy of projected vitamin D intakes compared with the EAR [39]. Fluid milk products and their alternatives were selected for modelling because they are widely consumed, nutrient-dense, and permitted for vitamin D fortification in Australia. Our modelling assumed equal bioactivity of the D vitamers; therefore, the relative contribution of these projected intakes may be greater if 25(OH)D in food has greater bioactivity than vitamin D [29,40,41]. Although more extensive modelling could include other foods (e.g., bread, orange juice, and breakfast cereals), only some dairy foods/alternatives, edible oil spreads, formulated beverages, and certain breakfast cereals are permitted for vitamin D fortification in Australia [17]. While the dietary intake data that were used in this study were collected in 2011–2012, it is unlikely that consumption practices have changed considerably since then. This, however, may apply to vitamin D supplements; vitamin D may be protective against acute respiratory infection [42,43,44], which has led to speculation that vitamin D sufficiency may reduce the severity of coronavirus disease 19 (COVID-19) infection [45] and may have led to increased supplement use. 

This study shows that fortification of fluid milks/alternatives with vitamin D at the current permitted level would produce a meaningful increase in vitamin D intakes, which could be of potential benefit to those with low vitamin D status. Nonetheless, this initial step would be insufficient to bring most of the population to the level of current intake recommendations in North America and Europe. In Australia, a review of nutrient reference values for vitamin D and representative data on the prevalence of vitamin D deficiency in certain population groups, such as children, are needed. However, a more extensive vitamin D fortification strategy would likely be needed to safely and effectively increase dietary vitamin D intake and improve vitamin D status in the Australian population.

## Figures and Tables

**Table 1:** Estimated vitamin D intakes ^1^ in a nationally representative sample of Australians at baseline and according to a vitamin D fortification scenario of 0.8 µg/100 mL in fluid milks/alternatives.

			Food Only ^2^		Food and Supplements ^3^	
Age Group, y	Sex	*n*	Baseline	Fortification Model	Baseline	Fortification Model
			Mean (95% Confidence Interval) (µg/day)			
≥2	All	12,153	2.95 (2.86, 3.04)	4.91 (4.81, 5.02)	5.27 (5.05, 5.48)	7.23 (7.01, 7.45)
≥2	Males	5702	3.16 (3.04, 3.27)	5.28 (5.13, 5.43)	4.58 (4.34, 4.82)	6.70 (6.45, 6.96)
≥2	Females	6451	2.74 (2.62, 2.87)	4.55 (4.40, 4.70)	5.95 (5.59, 6.31)	7.75 (7.39, 8.12)
2–3	Males	228	1.97 (1.62, 2.32)	4.67 (4.14, 5.20)	2.43 (2.01, 2.84)	5.13 (4.57, 5.69)
2–3	Females	236	1.83 (1.51, 2.15)	4.38 (3.88, 4.87)	2.19 (1.83, 2.55)	4.74 (4.23, 5.25)
4–8	Males	397	2.23 (2.04, 2.42)	4.35 (4.00, 4.70)	2.68 (2.41, 2.96)	4.80 (4.40, 5.20)
4–8	Females	392	2.20 (1.81, 2.58)	3.96 (3.53, 4.40)	2.73 (2.86, 3.18)	4.50 (4.02, 4.98)
9–13	Males	392	2.86 (2.54, 3.17)	5.05 (4.57, 5.54)	3.28 (2.83, 3.72)	5.47 (4.88, 6.07)
9–13	Females	395	2.88 (2.48, 3.28)	5.00 (4.94, 6.06)	3.25 (2.80, 3.70)	5.37 (4.78, 5.96)
14–18	Males	403	3.37 (2.95, 3.78)	5.50 (4.94, 6.06)	3.88 (3.34, 4.41)	6.01 (5.36, 6.67)
14–18	Females	369	2.28 (2.02, 2.54)	4.03 (3.56, 4.50)	3.36 (2.42, 4.29)	5.11 (4.16, 6.04)
19–30	Males	739	3.54 (3.13, 3.96)	5.76 (5.24, 6.28)	4.89 (4.10, 5.68)	7.10 (6.25, 7.96)
19–30	Females	853	2.75 (2.45, 3.05)	4.49 (4.06, 4.91)	5.12 (4.22, 6.02)	6.86 (5.91, 7.80)
31–50	Males	1669	3.12 (2.94, 3.30)	5.39 (5.14, 5.64)	4.80 (4.34, 5.25)	7.06 (6.57, 7.56)
31–50	Females	1896	2.78 (2.51, 3.06)	4.60 (4.28, 4.90)	5.86 (5.25, 6.47)	7.67 (7.05, 8.30)
51–70	Males	1341	3.30 (3.07, 3.53)	5.17 (4.90, 5.44)	4.93 (4.50, 5.36)	6.80 (6.35, 7.25)
51–70	Females	1565	2.90 (2.63, 3.17)	4.60 (4.30, 4.90)	8.07 (7.08, 9.06)	9.77 (8.77, 10.77)
≥71	Males	533	3.26 (2.87, 3.65)	5.14 (4.71, 5.58)	6.15 (5.14, 7.17)	8.03 (7.00, 9.06)
≥71	Females	745	3.04 (2.69, 3.41)	4.82 (4.42, 5.23)	9.50 (8.26, 10.74)	11.28 (10.02, 12.54)

^1^ We used Day 1 food and supplement consumption data from the 2011–2013 Australian Health Survey and vitamin D food composition data for vitamin D_3_, 25(OH)D_3_, vitamin D_2_, and 25(OH)D_2_. Data were weighted to the 2011–2012 Australian population. ^2^ *n* = 12,153. ^3^ *n* = 2039.

## Data Availability

Publicly available datasets were analysed in this study. These data can be found here: 2011–2012 National Nutrition and Physical Activity Survey https://www.abs.gov.au/statistics/microdata-tablebuilder/microdatadownload (accessed on 13 December 2021); AUSNUT 2011–2013 https://www.foodstandards.gov.au/science/Pages/default.aspx (accessed on 11 November 2021); and food composition data https://www.foodstandards.gov.au/science/monitoringnutrients/afcd/Pages/Data-provided-by-food-companies-and-organisations.aspx (accessed on 11 November 2021).

## References

[1] Dunlop E., Boorman J.L., Hambridge T.L., McNeill J., James A.P., Kiely M., Nowson C.A., Rangan A., Cunningham J., Adorno P. (2022). Evidence of low vitamin D intakes in the Australian population points to a need for data-driven nutrition policy for improving population vitamin D status. J. Hum. Nutr. Diet..

[2] Institute of Medicine (2011). Dietary Reference Intakes for Calcium and Vitamin D.

[3] National Health and Medical Research Council Nutrient Reference Values for Australia and New Zealand. https://www.nrv.gov.au/home.

[4] Nowson C.A., McGrath J.J., Ebeling P.R., Haikerwal A., Daly R.M., Sanders K.M., Seibel M.J., Mason R.S. (2012). Vitamin D and health in adults in Australia and New Zealand: A position statement. Med. J. Aust..

[5] Horton-French K., Dunlop E., Lucas R.M., Pereira G., Black L.J. (2021). Prevalence and predictors of vitamin D deficiency in a nationally-representative sample of Australian adolescents and young adults. Eur. J. Clin. Nutr..

[6] Malacova E., Cheang P., Dunlop E., Sherriff J., Lucas R.M., Daly R.M., Nowson C.A., Black L.J. (2019). Prevalence and predictors of vitamin D deficiency in a nationally-representative sample of adults participating in the 2011–2013 Australian Health Survey. Br. J. Nutr..

[7] Black L.J., Dunlop E., Lucas R.M., Pearson G., Farrant B., Shepherd C.C.J. (2020). Prevalence and predictors of vitamin D deficiency in a nationally representative sample of Australian Aboriginal and Torres Strait Islander adults. Br. J. Nutr..

[8] Australian Bureau of Statistics Vitamin D. https://www.abs.gov.au/articles/vitamin-d.

[9] Hill K.M., Jonnalagadda S.S., Albertson A.M., Joshi N.A., Weaver C.M. (2012). Top food sources contributing to vitamin D intake and the association of ready-to-eat cereal and breakfast consumption habits to vitamin D Intake in Canadians and United States Americans. J. Food Sci..

[10] Raulio S., Erlund I., Männistö S., Sarlio-Lähteenkorva S., Sundvall J., Tapanainen H., Vartiainen E., Virtanen S.M. (2017). Successful nutrition policy: Improvement of vitamin D intake and status in Finnish adults over the last decade. Eur. J. Public Health.

[11] Munasinghe L.L., Willows N.D., Yuan Y., Ekwaru J.P., Veugelers P.J. (2017). Vitamin D sufficiency of Canadian children did not improve following the 2010 revision of the Dietary Guidelines that recommend higher intake of vitamin D: An analysis of the Canadian Health Measures Survey. Nutrients.

[12] Ahluwalia N., Herrick K.A., Rossen L.M., Rhodes D., Kit B., Moshfegh A., Dodd K.W. (2016). Usual nutrient intakes of US infants and toddlers generally meet or exceed Dietary Reference Intakes: Findings from NHANES 2009–2012. Am. J. Clin. Nutr..

[13] Jääskeläinen T., Itkonen S.T., Lundqvist A., Erkkola M., Koskela T., Lakkala K., Dowling K.G., Hull G.L.J., Kröger H., Karppinen J. (2017). The positive impact of general vitamin D food fortification policy on vitamin D status in a representative adult Finnish population: Evidence from an 11-y follow-up based on standardized 25-hydroxyvitamin D data. Am. J. Clin. Nutr..

[14] Kiely M., Black L.J. (2012). Dietary strategies to maintain adequacy of circulating 25-hydroxyvitamin D concentrations. Scand. J. Clin. Lab. Investig..

[15] Cashman K., Kiely M. (2016). Tackling inadequate vitamin D intakes within the population: Fortification of dairy products with vitamin D may not be enough. Int. J. Basic Clin. Endocrinol..

[16] Calvo M.S., Whiting S.J. (2013). Survey of current vitamin D food fortification practices in the United States and Canada. J. Steroid Biochem. Mol. Biol..

[17] Food Standards Australia New Zealand Australia New Zealand Food Standards Code. https://www.foodstandards.gov.au/code/Pages/default.aspx.

[18] Herrick K.A., Storandt R.J., Afful J., Pfeiffer C.M., Schleicher R.L., Gahche J.J., Potischman N. (2019). Vitamin D status in the United States, 2011–2014. Am. J. Clin. Nutr..

[19] Ahmed M., Ng A.P., L’Abbe M.R. (2021). Nutrient intakes of Canadian adults: Results from the Canadian Community Health Survey (CCHS)–2015 Public Use Microdata File. Am. J. Clin. Nutr..

[20] Lips P., Cashman K.D., Lamberg-Allardt C., Bischoff-Ferrari H.A., Obermayer-Pietsch B., Bianchi M.L., Stepan J., El-Hajj Fuleihan G., Bouillon R. (2019). Current vitamin D status in European and Middle East countries and strategies to prevent vitamin D deficiency: A position statement of the European Calcified Tissue Society. Eur. J. Endocrinol..

[21] Spiro A., Buttriss J.L. (2014). Vitamin D: An overview of vitamin D status and intake in Europe. Nutr. Bull..

[22] U.S. Department of Agriculture Food and Nutrient Database for Dietary Studies 2013–2014: Factsheet. https://www.ars.usda.gov/ARSUserFiles/80400530/pdf/fndds/fndds_2013_2014.pdf.

[23] U.S. Department of Agriculture The USDA Food and Nutrient Database for Dietary Studies 2011–2013: Documentation and User Guide. https://www.ars.usda.gov/ARSUserFiles/80400530/pdf/fndds/fndds_2011_2012_doc.pdf.

[24] Health Canada Canadian Nutrient File-Users’ Guide. https://www.canada.ca/content/dam/hc-sc/migration/hc-sc/fn-an/alt_formats/pdf/nutrition/fiche-nutri-data/user_guide_utilisation-eng.pdf.

[25] Australian Bureau of Statistics Australian Health Survey: Users’ Guide, 2011–2013. https://www.abs.gov.au/ausstats/abs@.nsf/mf/4363.0.55.001.

[26] Bliss R.M. (2004). Researchers produce innovation in dietary recall. Agric. Res..

[27] Dunlop E., James A.P., Cunningham J., Strobel N., Lucas R.M., Kiely M., Nowson C.A., Rangan A., Adorno P., Atyeo P. (2021). Vitamin D composition of Australian foods. Food Chem..

[28] Food Standards Australia New Zealand AUSNUT 2011–2013. https://www.foodstandards.gov.au/science/monitoringnutrients/ausnut/Pages/default.aspx.

[29] Jakobsen J., Melse-Boonstra A., Rychlik M. (2019). Challenges to quantify total vitamin activity: How to combine the contribution of diverse vitamers?. Curr. Dev. Nutr..

[30] Boorman J.L., Baines J., Hambridge T.L., Abbey J.L. (2013). Food mapping in a total diet study. Total Diet Stud..

[31] Food Standards Australia New Zealand 25th Australian Total Diet Study. https://www.foodstandards.gov.au/publications/Documents/25thAustralianTotalDietStudy.docx.

[32] Food Standards Australia New Zealand FSANZ’s Dietary Exposure Assessment Computer Program. https://www.foodstandards.gov.au/science/exposure/Pages/fsanzdietaryexposure4439.aspx.

[33] Black L., Jacoby P., Nowson C., Daly R., Lucas R. (2016). Predictors of vitamin D-containing supplement use in the Australian population and associations between dose and serum 25-hydroxyvitamin D concentrations. Nutrients.

[34] Australian Government Department of Health (2022). Australian Register of Therapeutic Goods.

[35] Dunlop E., Kiely M.E., James A.P., Singh T., Pham N.M., Black L.J. (2021). Vitamin D food fortification and biofortification increases serum 25-hydroxyvitamin D concentrations in adults and children: An updated and extended systematic review and meta-analysis of randomized controlled trials. J. Nutr..

[36] Calvo M.S., Whiting S.J. (2006). Public health strategies to overcome barriers to optimal vitamin D status in populations with special needs. J. Nutr..

[37] Government of Canada Canada Gazette, Part I, Volume 152, Number 6: Regulations Amending Certain Regulations Made Under the Food and Drugs Act (Nutrition Symbols, Other Labelling Provisions, Partially Hydrogenated Oils and Vitamin D). https://canadagazette.gc.ca/rp-pr/p1/2018/2018-02-10/html/reg2-eng.html.

[38] Kiely M., Cashman K.D. (2018). Summary outcomes of the ODIN Project on food fortification for vitamin D deficiency prevention. Int. J. Environ. Res. Public Health.

[39] Carriquiry A.L. (1999). Assessing the prevalence of nutrient inadequacy. Public Health Nutr..

[40] Cashman K.D., Seamans K.M., Lucey A.J., Stöcklin E., Weber P., Kiely M., Hill T.R. (2012). Relative effectiveness of oral 25-hydroxyvitamin D3 and vitamin D3 in raising wintertime serum 25-hydroxyvitamin D in older adults. Am. J. Clin. Nutr..

[41] Ovesen L., Brot C., Jakobsen J. (2003). Food contents and biological activity of 25-hydroxyvitamin D: A vitamin D metabolite to be reckoned with?. Ann. Nutr. Metab..

[42] Jolliffe D.A., Jr C.A.C., Sluyter J.D., Aglipay M., Aloia J.F., Ganmaa D., Bergman P., Bischoff-Ferrari H.A., Borzutzky A., Damsgaard C.T. (2021). Vitamin D supplementation to prevent acute respiratory infections: A systematic review and meta-analysis of aggregate data from randomised controlled trials. Lancet Diabetes Endocrinol..

[43] Martineau A.R., Jolliffe D.A., Greenberg L., Aloia J.F., Bergman P., Dubnov-Raz G., Esposito S., Ganmaa D., Ginde A.A., Goodall E.C. (2019). Vitamin D supplementation to prevent acute respiratory infections: Individual participant data meta-analysis. Health Technol. Assess..

[44] Martineau A.R., Jolliffe D.A., Hooper R.L., Greenberg L., Aloia J.F., Bergman P., Dubnov-Raz G., Esposito S., Ganmaa D., Ginde A.A. (2017). Vitamin D supplementation to prevent acute respiratory tract infections: Systematic review and meta-analysis of individual participant data. BMJ.

[45] Pereira M., Damascena A.D., Azevedo L.M.G., Oliveira T.d.A., Santana J.d.M. (2020). Vitamin D deficiency aggravates COVID-19: Systematic review and meta-analysis. Crit. Rev. Food Sci. Nutr..

